# Real time flow with fast GPU reconstruction for continuous assessment of cardiac output

**DOI:** 10.1186/1532-429X-14-S1-W63

**Published:** 2012-02-01

**Authors:** Grzegorz T Kowalik, Jennifer A Steeden, Bejal Pandya, David Atkinson, Andrew M Taylor, Vivek Muthurangu

**Affiliations:** 1 Centre for Cardiovascular MR, UCL, Institute of Cardiovascular Science, London, UK; 2 UCL Department of Medical Physics & Bioengineering, Centre for Medical Image Computing, London, UK

## Background

Novel real-time phase contrast MR sequences allow assessment of flow during exercise. However, such sequences require a time-consuming reconstruction, which prevents continuous cardiac output monitoring during exercise.

Graphical processing units (GPU) offer the possibility of performing fast reconstruction of real-time MR-data. Such reconstructions would make continuous assessment of cardiac output during exercise possible and uncover new areas in cardiovascular monitoring. The aim of this project is to validate a novel real-time flow sequence with online GPU-based reconstruction.

## Methods

Twenty healthy volunteers underwent aortic flow assessment using spiral SENSE real-time PC-MR (12 interleaves, 4-fold SENSE acquisition, 43ms temporal resolution, 13980 frames, acquisition time 10 minutes). Aortic flow was measured continuously during a ramped cycle exercise (2-16W, increased every minute by 2W).

Real-time data was reconstructed using non-Cartesian iterative SENSE implemented using NVIDIA's CUDA technology on a networked computer equipped with a GTX-480 card. A bidirectional networking scheme was based on CORBA technology (omniORB library).

The absence of data dependencies makes it possible to divide the task into batches of 60 frames with incoming data being buffered on CPU memory. There were 3 reconstruction stages: data transmission onto the external machine, GPU processing and transmission of results to the scanner. The batched-reconstruction allowed for overlapping between these stages and thus efficient utilisation of the network connection and continuous reconstruction of data.

## Results

The new GPU implementation was tested and compared against our original CPU version. A single batch of 60 flow measurements was retrospectively reconstructed with both versions. GPU implementation showed negligible bias in stroke volume of -0.4ml and good limits of agreement -1.9 to 1.2ml. The batch reconstructed 7.7 times faster on the GPU than CPU (15.2 if time required for data transmission is excluded).

Total reconstruction time for the 13980 frames (including transmission and buffering) was ~626s. Thus, data was available for use ~9s after the scan finished (scan duration was ~617s). This is a 556 times speed-up compared to estimated CPU reconstruction (of ~83min).

**Figure 1:**
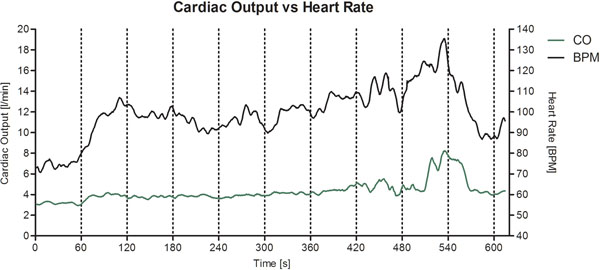


Fig 1 shows a representative plot of continuous heart rate and cardiac output changes during exercise in one volunteer.

## Conclusions

Continuous assessment of flow during exercise could provide a novel way of assessing hemodynamic responses in patients. Unfortunately, standard real-time MR sequences have long reconstruction times that take over one hour. We have shown that by performing a GPU reconstruction, the data can be clinically available within seconds of the acquisition finishing.

**Table 1:** Cooperation of iterative SENSE reconstruction implementations. Presented times are per iteration.

	CPU [ms]	GPU [ms]	CPU / GPU
FFT	143.83	36.97	3.9
Gridding	1337.37	29.24	46
Preconditioning	26.47	0.5	53
Iteration	3288.32	144.83	22.71

CPU vs GPU reconstruction.

